# Apical hypertrophic cardiomyopathy: pathophysiology, diagnosis and management

**DOI:** 10.1007/s00392-023-02328-8

**Published:** 2023-11-20

**Authors:** Jiangtao Li, Jing Fang, Yani Liu, Xiang Wei

**Affiliations:** 1 Division of Cardiovascular Surgery, Tongji Hospital, Tongji Medical College, Huazhong University of Science and Technology, 1095 Jiefang Ave., Wuhan, 430030 China; 2 Key Laboratory of Organ Transplantation, Ministry of Education, Wuhan, China; 3 NHC Key Laboratory of Organ Transplantation, Ministry of Health, Wuhan, China; 4 Department of Medical Ultrasound, Tongji Hospital, Tongji Medical College, Huazhong University of Science and Technology, 1095 Jiefang Ave., Wuhan, 430030 China

**Keywords:** Apical hypertrophic cardiomyopathy, Midventricular obstruction and cavity obliteration, Apical aneurysm, Septal myectomy

## Abstract

Since the first description of apical hypertrophic cardiomyopathy (ApHCM) in 1976, contrasting information from all over the world has emerged regarding the natural history of the disease. However, the recommended guidelines on hypertrophic cardiomyopathy (HCM) pay a cursory reference to ApHCM, without ApHCM-specific recommendations to guide the diagnosis and management. In addition, cardiologists may not be aware of certain aspects that are specific to this disease subtype, and a robust understanding of specific disease features can facilitate recognition and timely diagnosis. Therefore, the review covers the incidence, pathogenesis, and characteristics of ApHCM and imaging methods. Echocardiography and cardiovascular magnetic resonance imaging (CMR) are the most commonly used imaging methods. Moreover, this review presents the management strategies of this heterogeneous clinical entity. In this review, we introduce a novel transapical beating-heart septal myectomy procedure for ApHCM patients with a promising short-time result.

**Graphical abstract:**

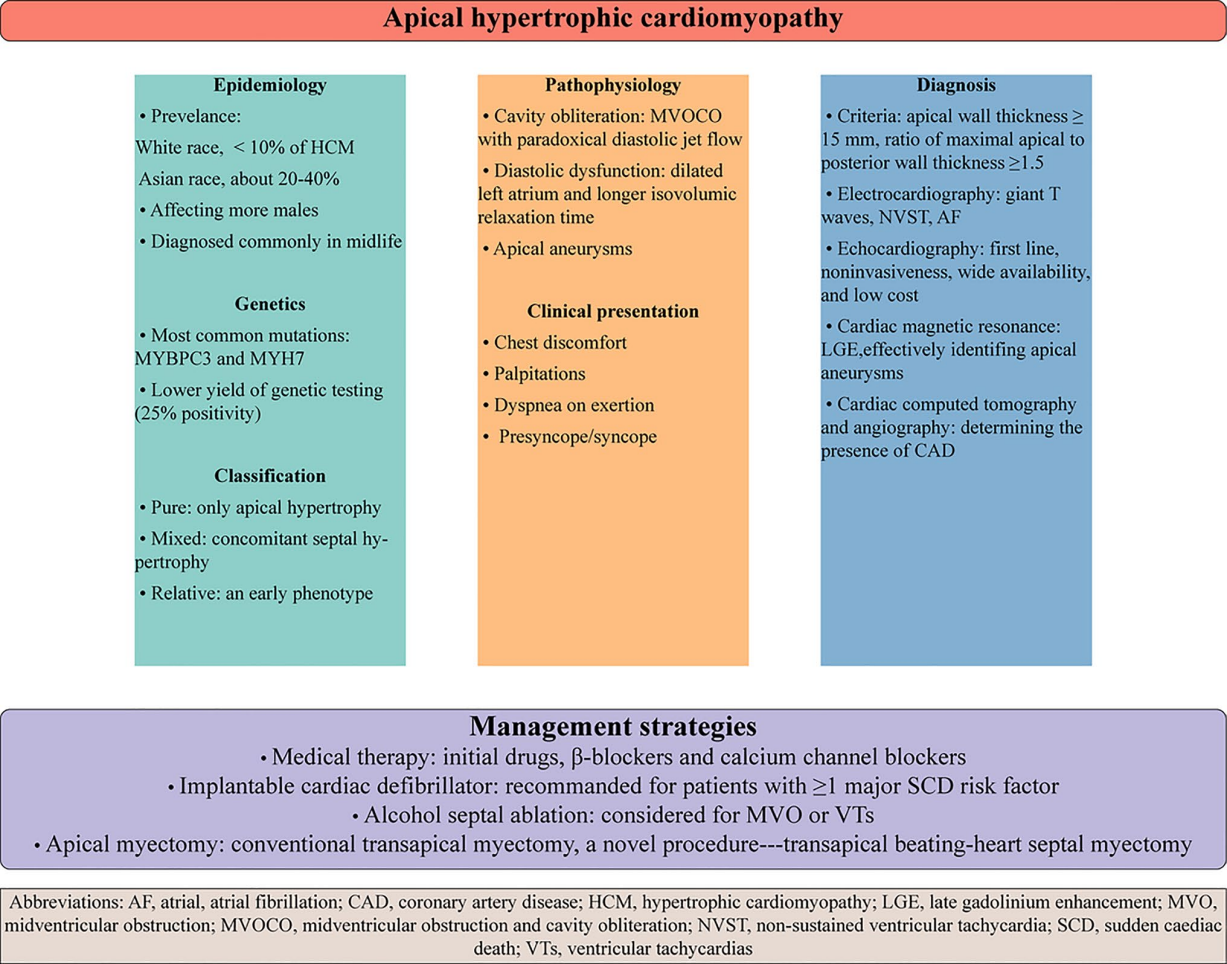

## Introduction

**Fig. 1:**
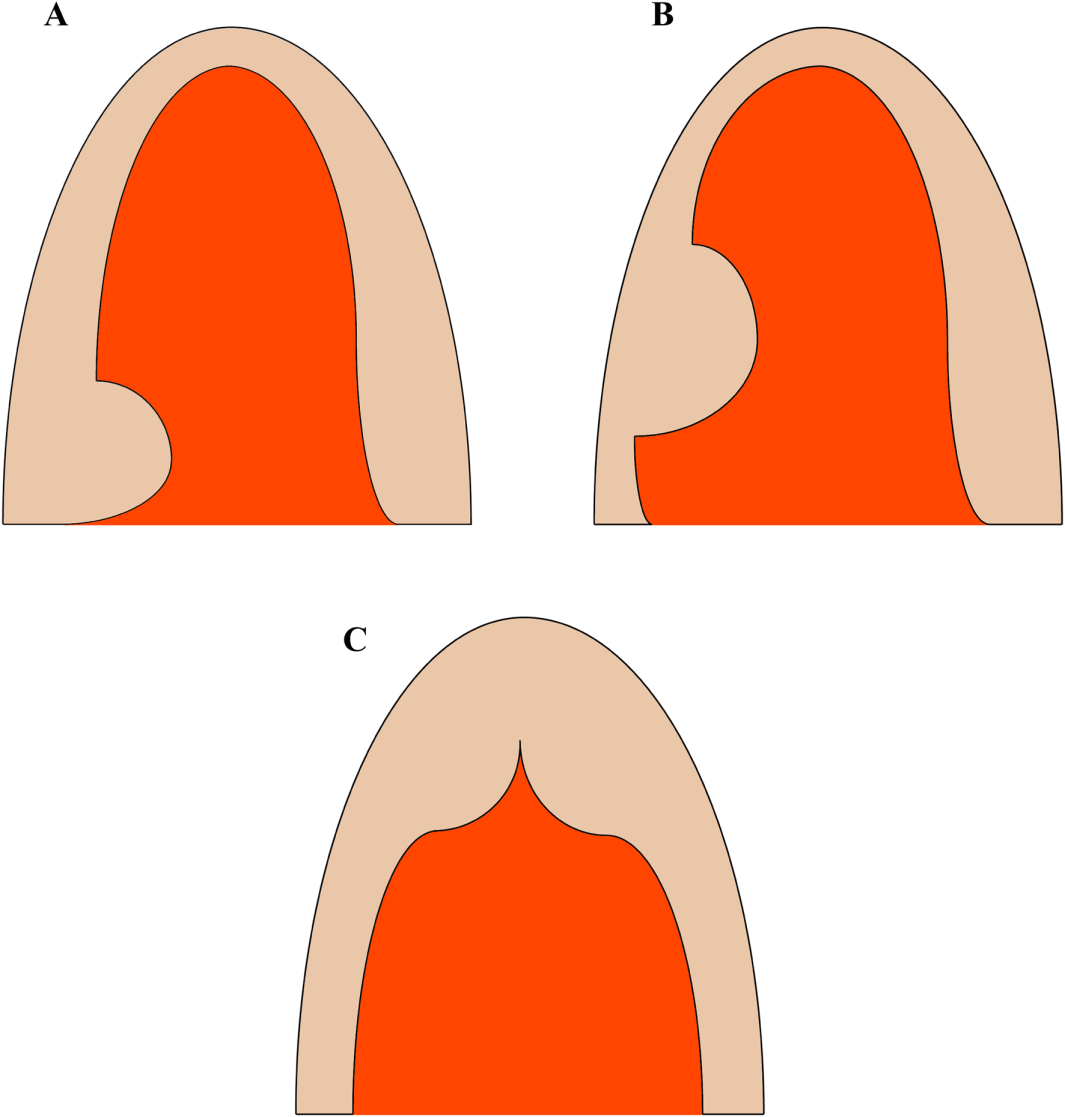
Variants of hypertrophic cardiomyopathy. Different morphologic types of hypertrophic cardiomyopathy: **A** basal, **B** midventricular, and **C** apical

Hypertrophic cardiomyopathy (HCM) is mostly an autosomal dominant disease characterized predominantly by the detection of left ventricular (LV) hypertrophy in the absence of another cardiac, systemic, or metabolic disease. According to the segments of LV hypertrophy, HCM can be classified into basal (also called classic HCM), midventricular, and apical (Fig. 1). Apical hypertrophic cardiomyopathy (ApHCM) is characteristic of hypertrophy predominantly involving the LV apex with giant negative T waves in the electrocardiogram and a “spade-like” configuration of the LV cavity on the LV ventriculogram. However, the current guidelines [1, 2] on HCM pay a cursory reference to ApHCM, without ApHCM-specific recommendations to guide the diagnosis and management. Thus, this review summarizes the epidemiology, pathophysiology, and clinical characteristics of ApHCM, while also highlighting knowledge of diagnostic techniques and management strategies. In this review, we introduce a novel transapical beating-heart septal myectomy procedure for ApHCM patients with a promising short-time result.

## Epidemiology

Hypertrophic cardiomyopathy is estimated to affect 1 out of 500 people. The ApHCM has a different prevalence in cohorts of patients with HCM, which is relatively higher in Asian ethnicity (Table 1). It is considered less common (8%) in Europe and North America with a majority (84%) of White race [3]. However, ApHCM may occur more frequently in Asian race, in whom it is seen in up to 40% of HCM patients (21% in China [4], 30% in Japan [5], and 38% in Korea [6]) of patients with HCM.

**Table 1:** Epidemiology of ApHCM in published studies

Study	Year published	Age, years	Male, *n* (%)	Ethnicity	Prevalence, *n* (%)
Eriksson et al. [38]	2002	46 ± 15	78 (74%)	White	–
Kitaoka et al. [79]	2003	45 ± 14	6 (60%)	White	10 (2.8%)
Kitaoka et al. [79]	2003	56 ± 14	15 (83%)	Asian	15 (15%)
Yang et al. [80]	2005	48 ± 14	113 (71%)	Asian	91 (36%)
Kubo et al. [5]	2009	61 ± 13	66 (83%)	Asian	80 (30%)
Binder et al. [34]	2011	61 ± 17	120 (62%)	White	210 (8%)
Moon et al. [6]	2011	61 ± 11	316 (70%)	Asian	454 (38%)
Yan et al. [36]	2012	52 ± 12	153 (74%)	Asian	208 (16%)
Kim et al. [81]	2013	60 ± 12	175 (72%)	Asian	243 (31%)
Klarich et al. [82]	2013	58 ± 17	120 (62%)	White	210 (8%)
Cai et al. [83]	2014	40 ± 15	45 (75%)	Asian	263 (13%)
Kim et al. [25]	2016	56 (49–64)	75 (88%)	Asian	85 (24%)
Neubauer et al. [3]	2019	51 ± 10	175 (78%)	White	224 (8%)
Yin et al. [4]	2022	57 ± 11	50(54%)	Asian	142 (21%)

Values are presented as numbers (percentages), mean ± standard deviation, or median (interquartile range)

*ApHCM* apical hypertrophic cardiomyopathy

ApHCM is worldwide in distribution and affects males more frequently than females, with male-to-female ratios typically 1.6 to 2.8:1 [7]. Most commonly diagnosed in midlife [3], the early- and late-onset expressions are also known to occur.

## Genetics

HCM is a genetic disorder of the myocardium, inherited in an autosomal dominant pattern with variable expressivity and age-related penetrance. So far, over 1500 mutations in 15 or more genes encoding the sarcomeric proteins and related myofilament elements have been reported in approximately half of the patients [8–10]. Mutations in the beta-myosin heavy chain (MYH7) and myosin binding protein C (MYBPC3) are most common, accounting for up to 50% of familial HCM patients [8, 11, 12]. About 5% of patients have at least two mutations and about 30% have genetically unexplained diseases.

Similar to classic HCM, although ApHCM involves genetic mutations (autosomal dominant inheritance pattern), implicating a role for genetics in the development of this morphological pattern of hypertrophy [13], this has been poorly explained. Genetic analyses based on studies of the small-sized population with ApHCM identified a predilection for ACTC1 (cardiac a-actin) mutations, indicating direct causality of ApHCM with cardiac actin mutations like Glu101Lys and association of ApHCM with ACTC E101K mutation [14, 15]. However, in the largest cohort of patients with genetic testing for HCM to date, the apical form was most commonly associated with mutations in MYBPC3 (6; 33%) and MYH7 (6; 33%), similar to the general HCM cohort [16]. In this study, the yield of genetic testing in ApHCM is relatively low (only 25% positivity).

## Classification

Morphologically ApHCM can be subclassified into three subtypes [17]: pure, mixed, and relative ApHCM (Supplementary Fig. 1). Pure AHCM presented with hypertrophy that was confined to the apex. Mixed ApHCM displayed both apical and septal hypertrophy but with the thickest apex. Finally, relative ApHCM is believed to be an early ApHCM phenotype. Individuals with relative ApHCM do not meet conventional diagnostic criteria for ApHCM but have similar imaging findings with the pure type. Relative ApHCM can be diagnosed when electrocardiography shows characteristic precordial T-wave inversion and cardiac imaging techniques show apical wall thickness exceeding basal wall thickness, although failing to reach the cutoff of wall thickness ≥ 15 mm [18].

**Fig. 2:**
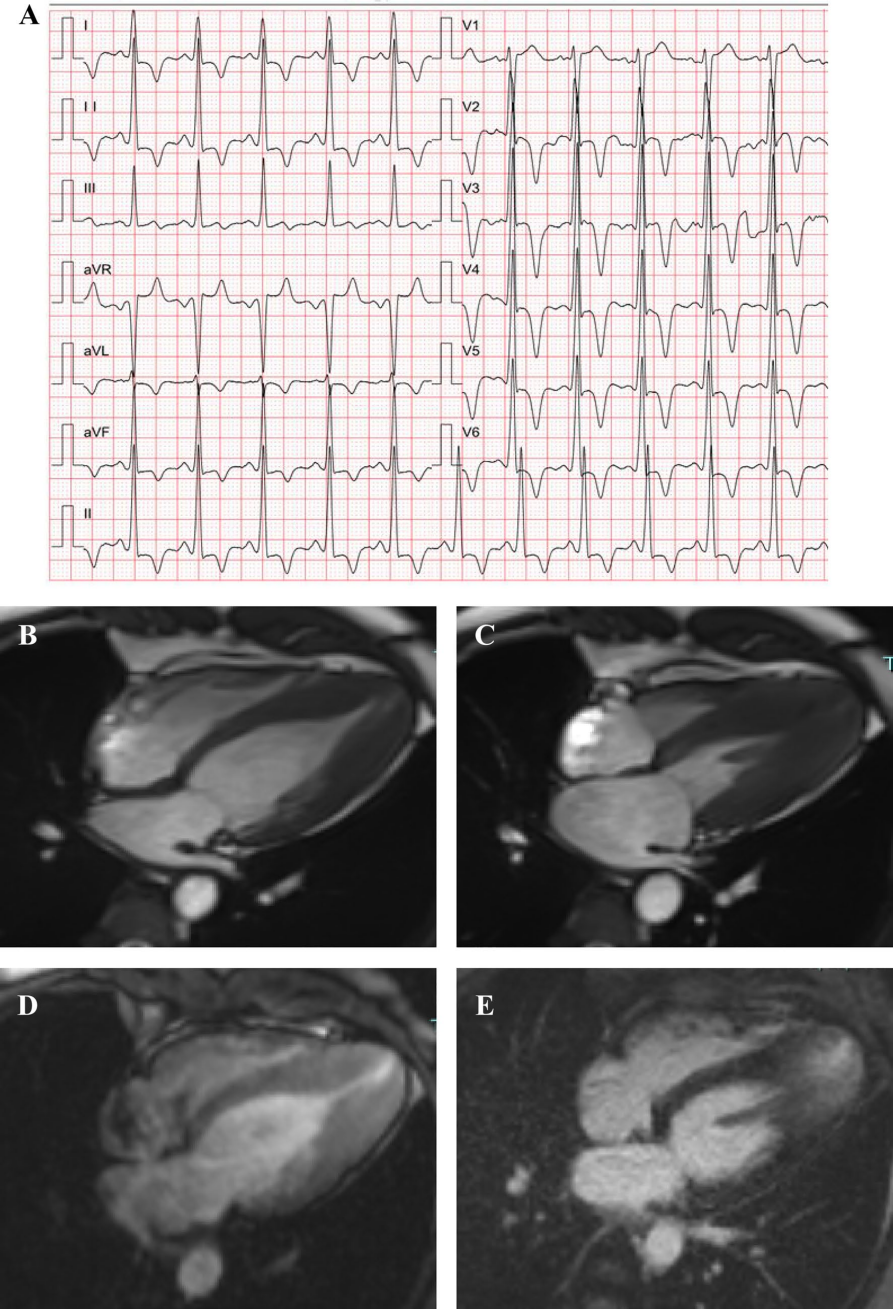
ECG and CMR in relative ApHCM. ECG demonstrates giant negative T-wave inversion in precordial leads and voltage criteria for LV hypertrophy (**A**). CMR demonstrates a thickened LV apex (13.5 mm) with relative but not absolute apical hypertrophy in diastole (**B**), apical cavity obliteration in systole (**C**), a small LV apical aneurysm (**D**), and the presence of LGE in the apex (**E**). *ApHCM* apical hypertrophic cardiomyopathy, *CMR* cardiac magnetic resonance imaging, *ECG* electrocardiogram, *LGE* late gadolinium enhancement, *LV* left ventricular

Relative ApHCM was originally considered entirely benign [17], but Flett et. al [18] suggested associated pathology with left atrial dilatation, apical obliteration, apical aneurysm, and myocardial scar (Fig. 2). In another study, given the absence of other causes of this abnormality, relative apical hypertrophy appeared to be the only explanation for giant T-wave (≥ 1 mV) inversion [19]. Thus, relative ApHCM may solely represent the early course of the disease that would with time progress to overt ApHCM and meet the conventional criteria.

## Pathophysiology

### Cavity obliteration

Apical cavity obliteration occurs usually in patients with ApHCM. The degree of apical cavity obliteration is measured by the ratio of obliteration to cavity, which is defined as the end-systolic length of apical obliteration to the end-systolic height of the LV cavity [20]. Apical hypertrophy is an ongoing process and causes cardiac function to deteriorate later on. Thus, the effect of apical obliteration can strengthen gradually as the LV cavity size decreases, which is proved by the ever-increasing adverse cardiovascular events identified during the follow-up [20]. In addition, severe apical cavity obliteration has been associated with myocardial ischemia at the obliterated area [21].

Hypertrophy involving the mid-ventricular segments can typically result in mid-ventricular obstruction (MVO) with cavity obliteration (MVOCO) [22], which is a complication of mixed rather than pure ApHCM (Supplementary Fig. 2). Indeed, MVO is related to the presence of apical aneurysms [23], the reduced survival and increased risk of ventricular arrhythmias [24]. In severe cases, MVOCO lasts into diastole and is associated with a paradoxical diastolic jet flow (PJF), which indicates an increased risk of thromboembolism, arrhythmias, and a worse prognosis [21].

### Diastolic dysfunction

LV wall thickening, especially of the apex, results in a decrease in the diastolic volume of the LV, consequently leading to the reduction of end-diastolic volume and cardiac output. Myocardial hypertrophy results in myocardial ischemia along with the formation of interstitial fibrosis, both responsible for increased chamber stiffness. Therefore, altered ventricular load with high LV filling pressures, nonuniformity in myocardial contraction and relaxation are common in patients with ApHCM, each of which could contribute to advanced diastolic dysfunction [6, 25]. ApHCM patients are reported to have diastolic dysfunction as evidenced by a dilated left atrium (LA) and longer isovolumic relaxation time, suggesting that local apical hypertrophy could affect global diastolic function [6, 26].

Diastolic dysfunction is the core of several clinical manifestations of ApHCM, including dyspnea, exercise intolerance, and pulmonary edema. With impairment in ventricular myocardial relaxation, greater dependency on the atrial systole for ventricular filling may occur, leading to dilatation of the LA, which increases the risk of atrial fibrillation (AF) episodes or other arrhythmias. Therefore, the LA enlargement could reflect the severity of diastolic dysfunction and predict poor prognosis in ApHCM.

### Apical aneurysms

The apical aneurysm is defined as a discrete, thin-walled, and dyskinetic or akinetic segment of the most distal portion of the LV [27]. The incidence of the apical aneurysm has been reported to account for approximately 2–5% of HCM [28–30], although it is a more common (20–30%) finding in the ApHCM subgroup [18, 25, 30]. A cue to their presence is the persistence of apical blood pooling distal to the point of apical systolic cavity obliteration. The aneurysm size is defined as the maximum transverse dimension measured by CMR or echocardiography in the four-chamber view at the end-systole and is further characterized as small (< 2 cm), medium (2–4 cm), and large (> 40 mm) [31].

There are only a few reports focusing on the dynamic process of apical aneurysm formation. In a recent study of 72 patients with ApHCM, the course of dynamic change of apical aneurysm formation was recorded by using at least two CMR examinations, indicating that the formation of apical aneurysm may follow four stages [32]. (1) It starts with apical systolic cavity obliteration or an apical slit due to the increased thickness of the apical wall. (2) Then the apical slit broadens in systole due to the combination of proximal obstruction of the hypertrophied segments and reduced contractile movement of the distal hypertrophy. (3) The apical slit subsequently develops into an apical outpouching. (4) Finally, the apical aneurysm forms. Yang et al. [32] suggested that the interval between the two stages was variable, but the mean interval from ApHCM to the development of apical aneurysm would take over 10 years, which was consistent with the findings of previous reports [33]. However, further studies are needed to identify a more detailed process of apical aneurysm formation.

The clinical course of ApHCM patients with apical aneurysms is variable but overall proved to be unfavorable [27]. Previous studies have shown that apical aneurysm is associated with a higher risk of adverse cardiovascular events, including cardiovascular death, ventricular tachycardia, and AF [28, 31]. Moreover, the apical aneurysm carries the risk of apical thrombosis and thromboembolic stroke [34]. Rowin et al. [28] reported that the appropriate ICD therapy rate for primary prevention was 4.0%/year in patients with apical aneurysm, about fivefold greater than the sudden cardiac death (SCD) event rate in those without apical aneurysm.

## Clinical characteristics

### Clinical presentation

The patients with ApHCM do not have any pathognomonic clinical symptoms and their main complaints are unspecific, leading to late diagnosis [35]. The most common presenting symptoms are chest pain, palpitations, dyspnea, as well as syncope [4]. In a series of 208 consecutive patients with AHCM, chest discomfort (defined as chest tightness or chest pain) was reported by 91.8% of patients, palpitations by 30.8%, dyspnea on exertion by 10.6%, presyncope/syncope by 7.2% [36]. Yin and colleagues [4] reported that 42.1% of ApHCM patients had a history of hypertension, 22.2% with heart failure, 13.5% with diabetes, and 7.1% with ischemic heart disease. ApHCM also occasionally manifests as morbid events such as atrial fibrillation, ventricular fibrillation, and sudden cardiac death [7, 37].

### Prognosis

The ApHCM was originally thought to have a favorable long-term prognosis. With a mean follow-up of 13.6 ± 8.3 years from presentation, the North American cohort of 105 mildly symptomatic patients with ApHCM showed low mortality of 10.5% (11/105) and annual mortality of 0.6% [38]. In another study from China, 3 (1.6%) of 208 patients with ApHCM died during a mean follow-up of 8.0 ± 3.5 years, and the annual mortality was 0.3% [5]. However, Klarich et al. [7] observed a cohort of 193 patients for more than 20 years and found a worse 20-year survival in patients with ApHCM versus expected for the normal population (47% versus 60%). Among a referral-based cohort of 126 patients with ApHCM [4], 34 (27%) experienced adverse events during a mean follow-up of 8.0 ± 3.0 years, including (1) cardiac death events (4.8%); (2) myocardial infarction unrelated to coronary artery disease (2.4%); (3) progressive heart failure with an increase of at least one NYHA functional class (13.5%); (4) appropriate implantable cardioverter-defibrillator (ICD) interventions for ventricular tachycardia or ventricular fibrillation (5.6%); (5) new-onset AF (6.3%); (6) and thromboembolic stroke (4.0%).

In an observational study of 208 Chinese patients, only age > 60 years, left atrial diameter > 36 mm, and NYHA class III were independently associated with increased risk of death [36]. A coexisting CAD was identified as a strong risk factor for survival in patients with ApHCM [39]. In addition, the distribution of hypertrophy was reported to influence survival; the mixed form and more severe hypertrophy were associated with a worse prognosis [5]. Further, patients with complete end-systolic cavity obliteration and apical aneurysm have been found to have higher cardiovascular morbidity [20, 27].

## Diagnosis

### Diagnostic criteria

ApHCM is described as an electrocardiographic pattern of giant negative T-waves (≥ 1 mV) together with a “spade-like configuration” of the LV cavity on the left ventriculography. [40] With advances in imaging techniques, the current definition mainly relies on demonstrating LV hypertrophy predominating in the LV apex, with the apical wall thickness ≥ 15 mm and a ratio of maximal apical to posterior wall thickness ≥ 1.5, based on echocardiography or CMR [38]. However, recent studies used the diagnostic criteria as inappropriate apical hypertrophy (loss of apical tapering and apical thickness exceeding basal thickness) but < 15 mm, and other characteristics (apical cavity obliteration, or apical aneurysm) [30], highlighting a novel trend of using a lower diagnostic cutoff.

### Electrocardiography

The trademark electrocardiographic finding for ApHCM is LV hypertrophy with giant T waves, which are defined as T-waves ≥ 1 mV in any electrocardiography lead. These giant T waves are dynamic and will change over time, or even disappear, as a part of the natural history of ApHCM [41]. The depth of T waves varies among patients, in a study of 208 ApHCM patients, Yan et al. [38] reported that 60 (28%) patients had giant T-waves, but only 11% of ApHCM patients had giant T waves in a series from Mayo Clinic [7]. Although giant T-waves in the setting of LV hypertrophy are considered pathognomonic for ApHCM, it is unlikely to rule out other causes of ST-T wave abnormalities, such as other types of HCM, coronary heart disease, medication effect (e.g., digoxin), and neurological diseases (e.g., subarachnoid hemorrhage). Considering the existence of numerous potential differential diagnoses, it is necessary to conduct a comprehensive evaluation through cardiac imaging to confirm the diagnosis of ApHCM.

Another suggested examination is ambulatory 24- or 48-h electrocardiography, which is crucial to detect non-sustained ventricular tachycardia (NSVT), AF, and ventricular fibrillation. Eriksson et al. [38] showed that Holter monitor recordings revealed NVST in 20 patients (23%) with ApHCM, which was found to be correlated with the presence of fibrosis. Several studies have reported that the prevalence of AF was 11–17% [4, 5]. LA enlargement secondary to LV diastolic dysfunction in patients with ApHCM can predict subsequent AF, which is prognostically adverse [42].

### Echocardiography

In contemporary clinical practice, transthoracic echocardiography (TTE) is the first-line imaging modality for the evaluation of ApHCM because of its noninvasiveness, wide availability, and low cost [43, 44]. A comprehensive echocardiographic examination should involve the assessment of (1) the distribution and magnitude of LV hypertrophy; (2) the presence of cavity obliteration and apical aneurysm; (3) obstruction at any level in the LV; (4) systolic and diastolic function; (5) and mitral apparatus and papillary muscle abnormalities [43–45]. ApHCM is diagnosed as having asymmetrical LV hypertrophy confined predominantly to the LV apex, an apical wall thickness ≥ 15 mm, and a ratio of apical to posterior wall thickness ≥ 1.5 [46, 47]. However, as the apical cap is the thinnest part of the LV, the cut-off value (15 mm) may be less in the presence of the accompanying findings [17, 48]. A lower threshold can be used to diagnose ApHCM when the clinical presentation and other imaging characteristics lend themselves to the diagnosis of ApHCM [30]. The apical wall thickness should be carefully assessed as foreshortening on segment measurements can lead to an overestimation of apical thickness by up to almost 30% [49].

Local dysfunction involves the apical segments, manifested as hypokinetic, akinetic, or even dyskinetic, especially in the presence of an apical aneurysm [34]. LV twist is significantly decreased due to a reduction in apical rotation, leading to the loss of early diastolic suction and eventually diastolic dysfunction in ApHCM [50]. In ApHCM patients with MVOCO, diastolic intraventricular gradient often exists, manifesting as a directed flow of apex to base during early diastole—the PJF phenomenon, [23] which is associated with a high risk of systemic embolism, perfusion abnormalities, and ventricular arrhythmias.

Because of frequent difficulties in visualizing the apical endocardium, 2D echocardiography may result in misdiagnosis, as it was reported that echocardiography failed to diagnose ApHCM in 31.7% of patients [36]. Therefore, there is a need for an alternative imaging technique, and contrast echocardiography has been recommended as an alternative when 2D images are suboptimal [51]. The administration of echocardiographic contrast allows the excellent visualization of LV morphology and the presence of an apical aneurysm.

### Cardiac magnetic resonance imaging

Another useful examination is CMR, which provides high-resolution images and may detect early ApHCM phenotypes better than echocardiography [17]. The advantage of CMR is the complete, unrestricted coverage of LV morphology [52], so it can accurately determine the site and extent of hypertrophy and the presence of apical aneurysm [44]. Currently, the use of CMR is standard practice for a comprehensive evaluation in patients with HCM, especially when ApHCM is suspected [22].

By utilizing the inherent magnetic properties of different tissues and the distribution of gadolinium-based contrast agents, CMR can accurately quantify and visualize the patterns of dense focal extracellular matrix deposition as seen in replacement fibrosis by late gadolinium enhancement (LGE). In several studies of ApHCM patients imaged with CMR, LGE is reported in up to 65–80% of cases [4, 31]. It is reported that LGE is strongly associated with clinical severity in terms of severe symptoms, disease progression, and poor prognosis [4, 53]. In addition, a recent study demonstrated that ApHCM patients with extensive LGE (≥ 15% of LV mass) tended to suffer adverse events compared with those with LGE < 15% [31].

CMR is particularly effective for delineating the presence of apical aneurysm, which is a prognostic indicator for ApHCM [54]. In a recent study, echocardiography (without contrast use) can only correctly identify 29% (9/31) of apical aneurysms in ApHCM patients, and most misdiagnosed cases were small aneurysms [31]. Apical aneurysms may present as dyskinesis or akinesis usually with transmural scarring on LGE, and are associated with adverse outcomes [55]. In addition, apical thrombi can be revealed with CMR, which may be overlooked with non-contrast echocardiography [28].

### Cardiac computed tomography and angiography

Although not used commonly, cardiac computed tomography (CT) may be considered for diagnosis when echocardiography is technically limited and CMR imaging is contraindicated or unavailable. Cardiac CT provides excellent spatial resolution allowing for clear definition of LV structure (including hypertrophy pattern, wall thickness, detection of subaortic membrane, and intracardiac thrombus) and function [54, 56]. Moreover, in ApHCM patients with symptoms or evidence of myocardial ischemia, coronary CT is recommended and can detect the stenosis of coronary arteries with high sensitivity and specificity [57, 58]. Coronary CT is recommended for patients with a low to intermediate risk of CAD, which may contribute to early diagnosis of patients presenting chest pain [59].

Coronary angiography is usually performed in patients who are scheduled for apical myectomy and have risk factors for coronary atherosclerosis. With the existence of extensive CAD, the surgical strategy would alter to apical myectomy combined with coronary artery bypass grafting.

## Management strategies

In the absence of large randomized trials, therapy in patients with ApHCM is largely based on the management of classic HCM to improve functional capacity, reduce symptoms, and prevent disease progression. However, due to the differences in pathophysiology and clinical characteristics between classic HCM and ApHCM, the management of ApHCM may differ to some extent from the classic HCM (Table 2).

**Table 2:** Phenotypic and management differences between classic HCM and ApHCM

	Classic HCM	ApHCM
ECG	Deep, narrow Q-waves in the lateral and inferior leads AF and NVST are less common	T-wave inversion, giant negative T-waves (≥ 1 mV) AF and NSVT are relatively common
Associated morbidity	LVOTO, SAM, and MR	Apical aneurysm and MVOCO
Annual mortality, %	0.5–1 in all HCM [61, 84]	0.3–3 [36, 82]
Management strategies		
Catheter ablation	Septal radiofrequency ablation of hypertrophied basal septum in symptomatic LVOTO [85] and VT ablation [86]	Ablation of VT in a few cases [73]
Alcohol septal ablation	Alcohol septal ablation of hypertrophied basal septum in symptomatic LVOTO	Potential role in symptomatic ApHCM with MVOCO Considered after the failure of VT catheter ablation
Surgery	Transaortic septal myectomy and a novel transapical beating-heart septal myectomy procedure, with improved symptoms and relief of LVOTO [87]	Transapical myectomy, with improved symptoms and superior survival [77] Apical aneurysm resection in persistent monomorphic VT Transapical beating-heart septal myectomy procedure, with enlarged LV cavity and increased stroke volume

*ApHCM* apical hypertrophic cardiomyopathy, *ECG* electrocardiogram, *HCM* hypertrophic cardiomyopathy, LVH left ventricular hypertrophy, *LVOTO* left ventricular outflow tract obstruction, *MR* mitral regurgitation, *MVOCO* mid-ventricular obstruction and cavity obliteration, *NSVT* non-sustained ventricular tachycardia, *SAM* systolic anterior motion, *VT* ventricular tachycardia

### Medical therapy

In ApHCM, beta-blockers are usually the initial drugs of choice and are often employed to reduce the prevalence of non-sustained ventricular arrhythmias. Their use is associated with (1) decreased heart rate response to exercise; (2) improved diastolic function; (3) relief of chest discomfort and dyspnea by reducing myocardial oxygen demand; and (4) decreased midventricular gradients with the existence of MVO. Despite these advantages, whether treatment with beta-blockers ultimately impacts outcomes in ApHCM patients remains undefined and needs to be verified in future studies. In general, non-vasodilatory beta-blockers are preferred to avoid exacerbating gradients in ApHCM patients with MVO [60].

The beneficial effects of verapamil and diltiazem are mediated by their negative inotropic and chronotropic mechanism, resulting in reduced chest pain, prolonged LV filling time in diastole, and improved myocardial perfusion [61]. Isolated refractory chest pain may be difficult to control if high doses of β-blockers or non-dihydropyridine calcium blockers are not aggressively used. The medication dosage should be titrated to effectiveness with monitoring for atrioventricular conduction block or bradycardia, especially in the combined usage of β-blockers and calcium channel blockers.

Low-dose loop or thiazide diuretics can be used to improve dyspnea and reduce volume overload in ApHCM patients. To prevent hypovolemia and hypotension, it is necessary to cautiously use any of these diuretics, usually by intermittent dosing as needed or chronic low-dose treatment.

Several studies aimed at the anti-fibrotic and antihypertrophic approaches targeting the renin–angiotensin–aldosterone system (RAAS), provide an additional treatment option for ApHCM. The benefit of RAAS inhibition on symptoms and mortality is assumed, and patients with heart failure and reduced LVEF are recommended to be treated with angiotensin-converting enzyme inhibitors or angiotensin II receptor blockers, in line with the 2021 ESC Guidelines for the management of chronic heart failure [62]. A small randomized, double-blind, placebo-controlled trial in twenty patients with non-obstructive HCM showed that losartan slowed the progression of myocardial hypertrophy and fibrosis in patients with non-obstructive HCM [63]. However, in a larger randomized study of 133 patients with obstructive or non-obstructive HCM, treatment with losartan had no effect on cardiac function or exercise capacity compared with placebo [64], and challenged the generally held view that angiotensin II receptor blockers reduced cardiac hypertrophy [65].

Mavacamten is a selective allosteric inhibitor of cardiac myosin ATPase specifically developed to target the underlying pathophysiology of HCM by reducing actin–myosin cross-bridge formation [66, 67], thereby reducing contractility and improving myocardial energetics [68]. Although mavacamten in patients with symptomatic non-obstructive HCM was associated with a significant dose-dependent reduction in N-terminal pro-B-type natriuretic peptide (NT-proBNP), there was no significant impact of mavacamten on symptoms or functional capacity [69]. In a more recent randomized phase 3 trial, mavacamten markedly improved exercise capacity, NYHA functional class, and health status in patients with obstructive HCM [70]. Therefore, this agent might be only beneficial for ApHCM patients with MVO, but further studies are needed to confirm the benefit.

### Implantable cardiac defibrillator

There are currently no randomized studies to guide the insertion of an implantable cardiac defibrillator (ICD) specifically for ApHCM. Over the past decades, a large number of studies have focused on identification of the clinical risk markers. Maron et al. [71] have recently evolved the American Heart Association guidance for ICDs by proposing new criteria for HCM patients, which includes new high-risk markers such as systolic dysfunction (EF < 50%), extensive LGE on CMR occupying ≥ 15% of LV mass by quantification or visual estimation, and the LV apical aneurysm of any size [71]. This risk stratification seems more sensitive than the ESC guidance [2] in predicting patients with ApHCM under the risk of SCD and indicates significant progress in understanding more personalized risk factors.

The current SCD risk markers, obtained from personal and family history, and noninvasive examinations (including echocardiography, ambulatory electrocardiogram, and CMR), can be used to estimate the risk level of ApHCM patients and to determine those most likely to benefit from primary prophylaxis of ICD therapy. It is reasonable to implant an ICD for ApHCM patients with ≥ 1 major SCD risk factor [1], including (1) family history of HCM-related SCD; (2) massive LV hypertrophy; (3) unexplained syncope; (4) NSVT on the ambulatory monitor; (5) LV systolic dysfunction (EF < 50%); (6) Extensive LGE on CMR imaging; (7) LV apical aneurysm (Table 3). Although the potential risk factors (MVOCO, PJF, AF, etc.) are not currently supported by a large body of evidence linking the risk factor to sudden death, these serve in the risk arbitration of SCD in ApHCM.

**Table 3:** Major risk factors for sudden cardiac death in patients with ApHCM

Major risk factors	Description
Family history of HCM-related SCD	Family history of SCD judged to be definitively or likely caused by HCM in ≥ 1 first- or other close relatives ≤ 50 years [61]
Massive LVH	Maximal wall thickness ≥ 30 mm in any segment by echocardiography or CMR imaging [84]; consideration also given to borderline thicknesses of ≥ 28 mm at the discretion of treating cardiologist [85]
Unexplained syncope	≥ 1 unexplained episode with acute transient loss of consciousness, non-neuro cardiogenic (vasovagal) etiology, and especially when episodes within 6 months of evaluation [73, 86]
NSVT	Defined as ≥ 3 consecutive ventricular beats at ≥ 120 beats per minute lasting < 30 s during ambulatory electrocardiography monitoring [77, 87], with greater weight when associated with another risk marker, particularly LGE [85]
LV systolic dysfunction	Systolic dysfunction with EF < 50% by echocardiography or CMR imaging [21]
Extensive LGE	Diffuse and extensive LGE on CMR imaging, representing myocardial fibrosis, comprising ≥ 15% of LV mass (quantified or by visual estimation) [22]
LV apical aneurysm	Defined as a discrete, thin-walled, dyskinetic/akinetic segment of the most distal LV chamber, independent of size, with associated contiguous regional scarring [23]

*CMR* cardiac magnetic resonance imaging, *EF* ejection fraction, *LGE* late gadolinium enhancement, *LV* left ventricular, *LVH* left ventricular hypertrophy, *NSVT* non-sustained ventricular tachycardia, *SCD* sudden cardiac death

### Alcohol septal ablation and surgery

Unlike classic HCM, the absence of LV outflow tract obstruction can render alcohol septal ablation (ASA) in ApHCM unwarranted. However, some case studies have highlighted its potential role in symptomatic patients with MVO, as it may improve symptoms and reduce intraventricular gradients [72]. Therefore, in ApHCM patients with MVO and at high risk for surgery, ASA is considered in the hands of experienced surgeons. In addition, it was reported that ASA eliminated VT in patients with ApHCM after catheter ablation failed to abolish VTs [73]. As ASA may produce substantial infarction and harm ventricular function, it should be limited to highly selected patients.

According to the 2020 AHA/ACC guideline, apical myectomy can be performed by experienced surgeons at comprehensive centers in highly selected ApHCM patients with severe symptoms and lifestyle limitations (diastolic heart failure, NYHA class 3–4 dyspnea) despite maximal medical therapy, and with preserved EF and small LV cavity [1]. Transapical myectomy has been reported to improve functional status and enlarge the LV cavity as well as stroke volume [74]. In brief, the operation is performed through a median sternotomy with cardiopulmonary bypass. The apical incision is made lateral to and far enough from the left anterior descending coronary artery so that closure will not compromise the vessel. After identification of the papillary muscles, initial myectomy is performed along the ventricular septum and additional resection along the free wall can be performed to enlarge the LV cavity. In addition, the prominent papillary muscles can be shaved to further increase LV volume. If an apical aneurysm is present, it will be repaired. The apical incision and its closure produce an area of akinesis, but this is tiny and does not result in significant systolic dysfunction [75]. Concomitant transaortic myectomy is performed in those with symptomatic MVO, as it may reduce intraventricular gradients and improve heart failure symptoms [76].

**Fig. 3:**
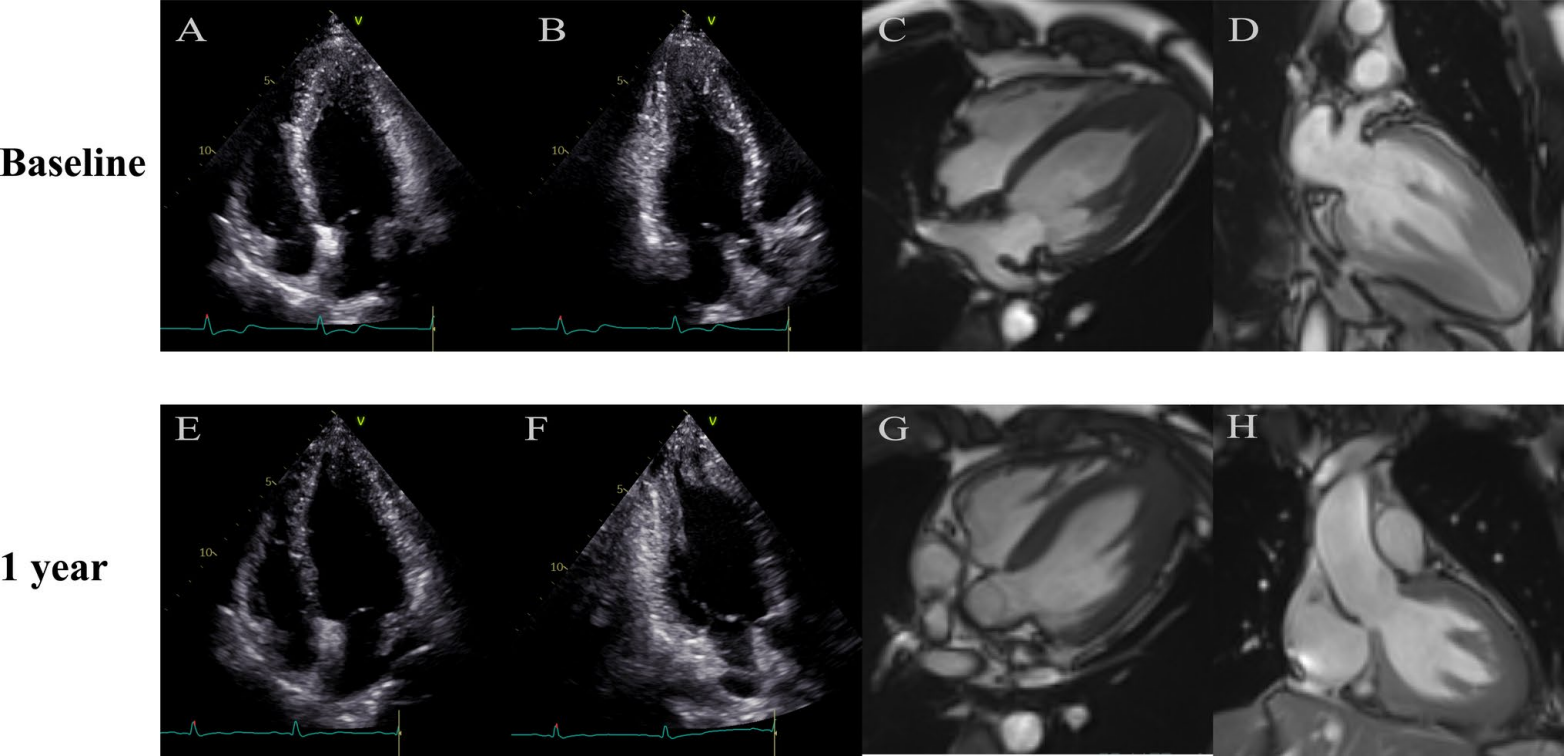
Transthoracic echocardiography (**A**, four-chamber; **B**, two-chamber) and cardiac magnetic resonance (**C**, four-chamber; **D**, two-chamber) in a patient with ApHCM at baseline. Note the increased thickness of the apex and small end-diastolic volume of the LV. Transthoracic echocardiography (**E**, four-chamber; **F**, two-chamber) and cardiac magnetic resonance (**G**, four-chamber; **H**, two-chamber) in the same patient one year after TA-BSM. Note the significantly reduced thickness of the apex and the increased end-diastolic volume of the LV. *ApHCM* apical hypertrophic cardiomyopathy, *LV* left ventricle, *TA-BSM* transapical beating-heart septal myectomy

In a recent study from the Mayo Clinic, 113 symptomatic patients with ApHCM and advanced heart failure underwent transapical myectomy [77], 76% (32/41) of patients reported improvement in general health during a median follow-up of 5.2 (3.2–10.1) years. The survival of patients receiving apical myectomy appears better than those with HCM listed for cardiac transplantation, with the overall estimated 1-, 5-, and 10-year survivals of 96, 87, and 74%, respectively [77]. In addition, a novel transapical beating-heart septal myectomy (TA-BSM) procedure has been performed in obstructive HCM patients through the left intercostal incision with no need for cardiopulmonary bypass [78]. In a recent clinical trial, 46 (97.9%) of 47 patients with obstructive HCM showed improved functional status and significant relief of obstruction at 3 months follow-up [78]. Therefore, patients with ApHCM may also benefit from the TA-BSM procedure. The surgical results of ApHCM patients were satisfactory with significantly enlarged LV cavity (Fig. 3) and increased stroke volume. These preliminary findings, however, remain to be confirmed over a longer follow-up period in a larger patient population.

## Conclusions

ApHCM is a complex subset of HCM that is highly heterogeneous in its clinical and pathological profile. ApHCM presents special characteristics in epidemiology, pathophysiology, diagnosis, and therapy compared with more commonly detected and better understood classic HCM. Although ApHCM is relatively rare, patients with non-specific symptoms, such as chest pain, dyspnea on exertion, or syncope, should be carefully examined for the presence of ApHCM. Contemporary echocardiography provides certainty to the morphology of ApHCM that characterizes the disease phenotype, including MVOCO, diastolic dysfunction, and apical aneurysm. In addition, CMR can accurately detect apical aneurysms and identify regions of LGE. Similar to classic HCM, patients with ApHCM should undergo SCD risk assessment to determine whether to implant ICD. Transapical myectomy can be performed in a selected group of patients with good long-term results. In addition, the TA-BSM procedure is a novel therapy for ApHCM patients with minimal trauma and favorable early outcomes. These preliminary findings, however, remain to be confirmed over a longer follow-up period in a larger patient population.

## Data Availability

The data underlying this article will be shared on reasonable request to the corresponding author.
